# MicroRNA expression data of pluripotent and somatic cells and identification of cell type-specific MicroRNAs in pigs

**DOI:** 10.1016/j.dib.2020.106563

**Published:** 2020-11-21

**Authors:** Jong-Nam Oh, Dongchan Son, Kwang-Hwan Choi, Jae Yeon Hwang, Dong-Kyung Lee, Seung-Hun Kim, Mingyun Lee, Jinsol Jeong, Gyung Cheol Choe, Chang-Kyu Lee

**Affiliations:** a Department of Agricultural Biotechnology, Animal Biotechnology Major, and Research Institute for Agriculture and Life Sciences, Seoul National University, Seoul 08826, Korea; b Department of Cellular and Molecular Physiology, Yale School of Medicine, New Haven, CT 06510, USA; c Designed Animal and Transplantation Research Institute (DATRI), Institute of Green Bio Science and Technology, Seoul National University, Pyeongchang 25354, Korea

**Keywords:** MicroRNA (miRNA), Pig, Pluripotency, Blastocyst, Embryonic stem cell-like cells, Embryoid body, Fetal fibroblasts

## Abstract

Typical models of pluripotency, humans and mice, have been used to analyse the characteristics of pluripotent stem cells. However, these species exhibit molecular differences in many aspects. With similar physiology and genomics as humans, pigs are promising model for the research of pluripotency. The data of porcine pluripotent cells would be helpful in understanding the molecular network of human pluripotency. Pluripotent cells of humans and mice exhibit specific MicroRNA (miRNA) expression patterns to maintain the pluripotent state. Information about miRNA expression in pig pluripotent cells is not sufficient, so we analysed miRNAs in pluripotent (blastocysts and ES-like) and somatic cell samples (PEB and PFF). We screened cell-type specific miRNAs and identified their target genes. Functional annotation of the target genes was also conducted. Our data may facilitate miRNA-based induction and maintenance of the pluripotent state of porcine cells and provide support to fill the gap between the pluripotency networks of humans and mice.

## Specifications Table

**Table:** 

Subject	Bioinformatics
Specific subject area	MicroRNA (miRNA) sequencing data and target prediction
Type of data	RNA sequencing data, Table, Figure
How data were acquired	Samples were prepared as decribed below. - Blastocysts; Maturated oocytes were electroactivated with an electric pulse and cultured in 7 days. - Pig fetal fibroblasts; Cells were obtained from 30-day-old fetuses. - Embryonic stem cell-like (ES-like) cells; Expanded blastocysts on day7 were used to derive ES-like cells. - Embryoid bodies; ES-like cell colonies were detached and suspension cultured in hanging-drops. Next generation sequencing using HiSeq2500 and additional analysis. - Adapters were applied on miRNAs isolated from samples. - Sequencing was performed using HiSeq2500 (Illumina) - Quality of the reads were checked and adapters were trimmed out. - All small RNAs were quantified. - Reads were normalized within samples and categorized using miRDeep2. - Sample-specific miRNAs were listed and their target genes were predicted.
Data format	Raw data in FASTQ file, Filtered and Analysed
Parameters for data collection	Sequencing reads were quality checked and miRNAs were filtered by length from the reads. Reads that were uploaded on miRDeep2 based in miRBase were categorized into ‘reported’ and the other sequences were defined as ‘novel’ miRNAs. Expression levels of each miRNAs were normalized among samples. Gene ontology (GO) and Kyoto Encyclopedia of Genes and Genomes (KEGG) terms were used for target prediction analysis.
Description of data collection	Small RNAs were extracted from cell line and blastocyst samples and used to prepare RNA libraries. Sequencing reads were produced from RNA libraries using HiSeq2500 (Illumina) and miRNAs were filtered from the whole reads. All miRNAs were categorized as reported or novel miRNAs using miRDeep2. Sample or pluripotency specific miRNAs were sorted and used on target prediction.
Data source location	Institution: Seoul National University City/Town/Region: Seoul Country: Korea
Data accessibility	With the article and on a public repository Repository name: GEO database of NCBI Data identification number: GSE152934 Direct URL to data: https://identifiers.org/geo:GSE152934 Repository name: figshare Direct URL to data: https://doi.org/10.6084/m9.figshare.12622721.v3

## Value of the Data

• We present microRNA profiles of pluripotent and non-pluripotent cells in pigs. The list of miRNAs can provide a standard for the study of pluripotency and it can be utilized in many directions in the field of stem cells.
• Our data will help researchers who need pig-specific criteria to establish pluripotent cells. The list of pluripotent-specific miRNAs can be used to evaluate the pluripotent status of established cell lines, also can be used to support the induction of pluripotent cells from diverse origins.
• miRNAs on our data can be used directly to induce pluripotent cells, also they can be used to identify the status of cells. Our data will broaden the insight into the pluripotent status of cells in pigs.

## 1 Data Description

Sequencing data was generated by HiSeq2500. Cell type-specific miRNAs were determined using normalized expression levels. Raw data files were uploaded on NCBI website.

https://identifiers.org/geo:GSE152934

Datasets and figures are uploaded on figshare.

https://doi.org/10.6084/m9.figshare.12622721.v3

### 1.1 Sample preparation and miRNA extraction

Blastocysts (BLs) were generated by parthenogenetic activation in vitro as described in our previous works [1]. The embryos produced in vitro were stored at −80 °C until use. Dataset 2 contains sample and sequencing information. Pig fetal fibroblasts (PFFs) were cultured, and porcine embryoid bodies (PEBs) were derived from embryonic stem cell-like (ES-like) cells as previously described [2]. Extraction of miRNA was conducted using a mirVANA miRNA Isolation kit according to the manufacturer's instructions (Invitrogen, Carlsbad, CA).

### 1.2 Quality control and assessment of miRNA expression

**Fig. 1:**
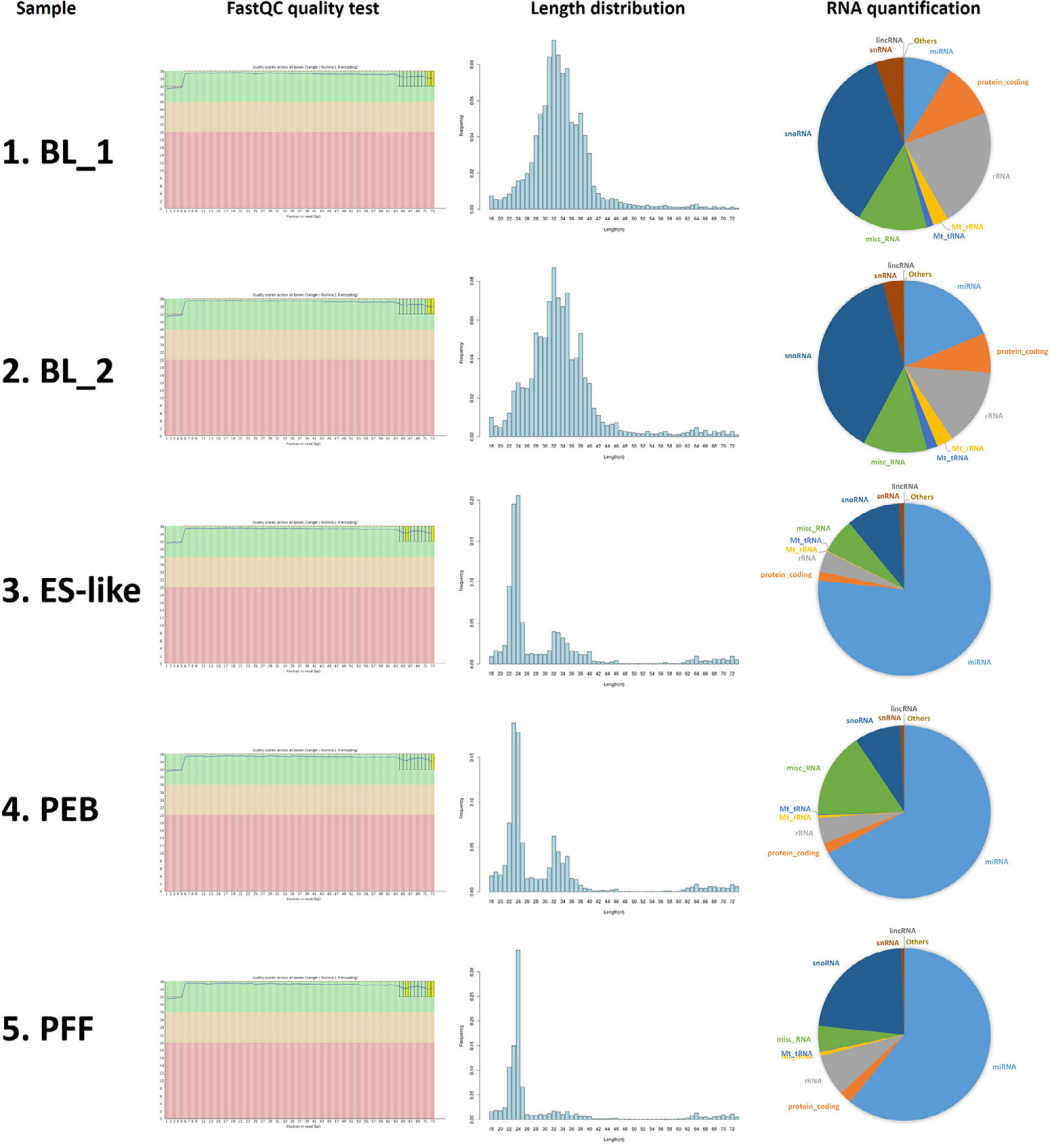
Quality control data of samples. (BL_1, BL_2; porcine day 7 blastocysts, PES; porcine embryonic stem cell, PEB; embryoid bodies from PES, PFF; porcine fetal fibroblasts) Result of FastQC quality test, length distribution of read and RNA ratio of each class were presented.

**Fig. 2:**
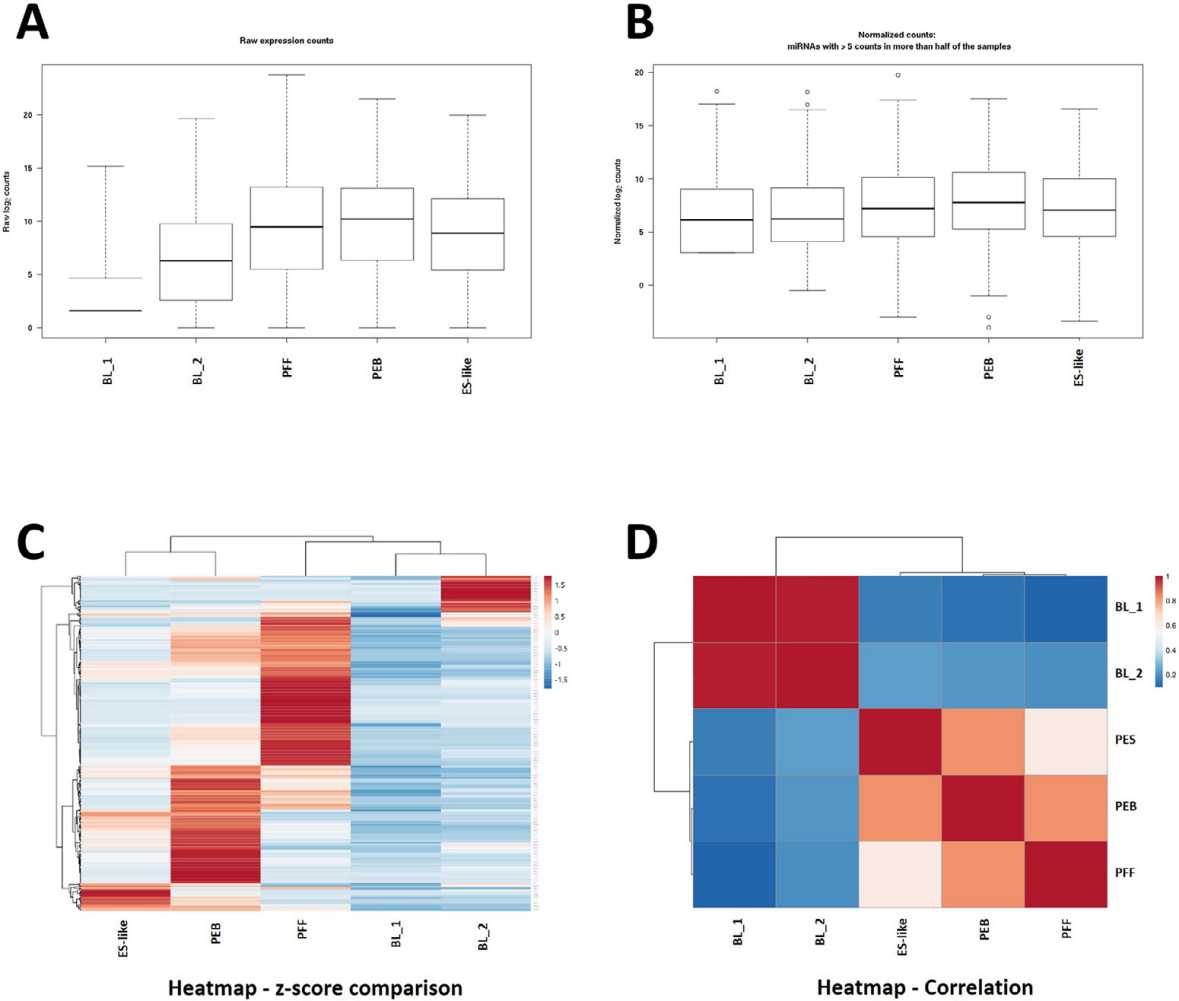
Normalization of samples and heatmaps for sample comparison A. Raw count of miRNA expression in each sample. B. Normalized miRNA expression level of samples. C. Heatmap for z-score comparision. D. Heatmap of correlation among samples.

HiSeq2500 (Illumina) was used for RNA sequencing (Dataset 1). Raw data from sequencing (GSE152934) was used for following analysis. To exclude low quality reads, sequenced reads that were shorter than 17 base pairs or lacked adaptor sequences were filtered out. Less than 10% of the reads were sifted out in every sample. Adaptor sequences in the remaining reads after filtering were trimmed out by Cutadapt. According to the FastQC test, the quality of the samples was sufficient for further analysis. The quality of the filtered reads was checked based on their length distribution (Fig. 1). Length distributions of the cultured cells showed steep peaks at a length of 23–24 nucleotides, but wide peaks were observed at a length of 32 nucleotides in the BL samples. All captured small RNAs were grouped into RNA categories using ENSEMBL/GENCODE annotations (Fig. 1). Ratios of miRNAs to whole small RNAs were 8.8% in BL_1, 18.7% in BL_2, 76.7% in ES-like cells, 67.0% in PEBs and 60.8% in PFFs. The screened miRNAs were classified as reported or novel miRNAs, and their expression levels were normalized (Dataset 3, Fig. 2A and B). Tag Count Comparison was used as the normalization method [3].

### 1.3 Comparative analysis miRNAs and target gene prediction

**Fig. 3:**
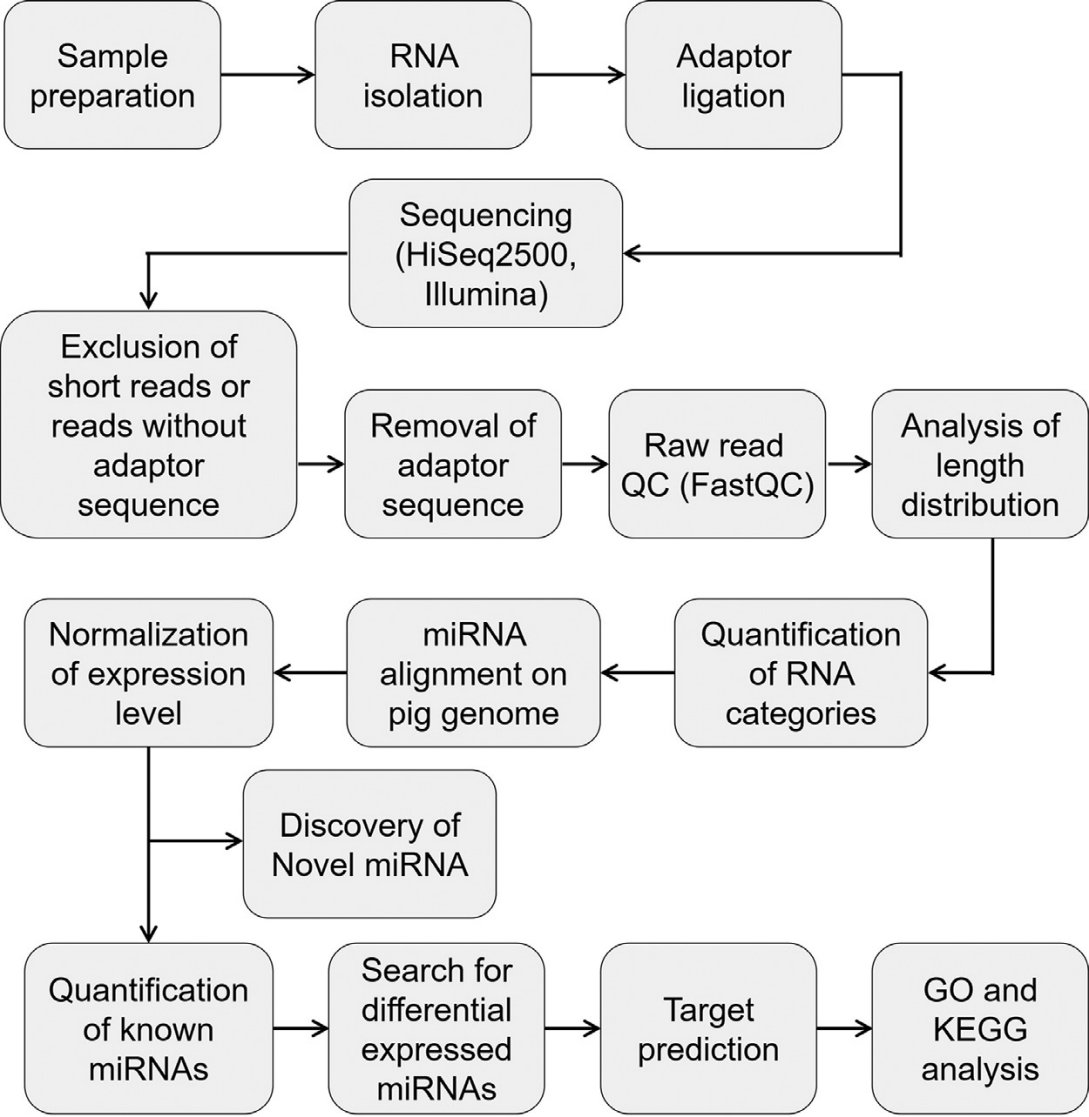
Workflow of miRNA-sequencing and data analysis.

Z-scores (Fig. 2C) and correlation coefficients (Fig. 2D) of normalized miRNA expression levels between the samples were calculated and are presented in heatmaps (sheet 1 of Dataset 2). The heatmaps show that the expression patterns of miRNAs in blastocysts and ES-like cells are clustered. In many NGS and omics studies, a 2-fold difference is used as a standard to compare targets between samples. We screened miRNAs that were specifically up- or downregulated more than 2-fold in each cell type and pluripotent cells (blastocysts and ES-like cells) or differentiated cells (PEBs and PFFs) (Dataset 3). The comparison conditions are described in sheet 3 of Dataset 3. A total of 72, 28, 19 and 39 miRNAs were filtered from BLs, ES-like cells, PEBs and PFFs, respectively. In addition, 15 and 22 miRNAs were filtered from pluripotent and differentiated cells, respectively (sheet 3 of Dataset 4). Target genes of the screened miRNAs were predicted (sheet 1 of Dataset 4). Functional annotation was performed, and the GO terms and KEGG pathways are listed on sheets 2–9 of Dataset 4. Tools and references for data analysis are described in the Supplementary document. A diagram of the overall workflow is shown in Fig. 3.

**Table:** Tools

Tools			
Name	Version	Description	
FastQC	0.10.1	Checks quality of the sequence data	
Cutadapt	1.10	Finds and removes adapter sequences	
miRDeep2	2.0.0.7	A software package for identification of novel and known miRNAs based on deep sequencing data	
Bowtie	1.1.1	An ultrafast, memory-efficient short read aligner	
Samtools	1.2	An umbrella organization encompassing several groups working on formats and tools for next-generation sequencing	
HTSeq	0.6.1p1	Counts reads in features (for quantification)	
VCFTools	v0.1.11	Can be used to perform operations on VCF files	
edgeR	3.10.2	Empirical analysis of digital gene expression data in R	
TargetScanHuman	7.2	Predicts target genes of miRNAs	

References			

Name	Version	Description	Website

Genome build	Ensembl 72 (S_scrofa)	Fasta format sequences of genome	http://jun2013.archive.ensembl.org
Geneset	Ensembl 72 (S_scrofa)	Gtf format file of reference release set	http://www.ensembl.org http://www.gencodegenes.org
Mature	21	Fasta format sequences of all mature miRNA sequences	http://mirbase.org/ftp.shtml
Hairpin	21	Fasta format sequences of all miRNA hairpins	http://mirbase.org/ftp.shtml
Genome coordinates	21	Gff3 format file of miRNA coordinates	http://mirbase.org/ftp.shtml

## 2 Experimental Design, Materials and Methods

### 2.1 Animal care

A pregnant sow was purchased from a local animal farm. The sow was taken care of exclusively by the farm and sacrificed 30 days after artificial insemination at a nearby slaughterhouse under approval by the Korean government.

### 2.2 Preparation of in vitro produced embryos

Production of embryos was conducted according to our previous report [1]. The ovaries of prepubertal gilts were collected from a local slaughterhouse. Follicular fluid and cumulus-oocyte-complexes (COCs) were aspirated from the ovaries using an 18-gauge needle. Sediments were washed with TLH-PVA medium, and selected COCs with compact cumulus were cultured in TCM199 medium (Life Technologies, Carlsbad, CA, USA) supplemented with 10  ng/mL epidermal growth factor, 1  mg/mL insulin, and 10% porcine follicular fluid for 44 hours at 39.8 °C at 5% CO2 and 100% humidity. The COCs were treated with gonadotropins (equine chorionic gonadotropin and human chorionic gonadotropin, Intervet, Cambridge, UK) for the first 22 h and then matured in the absence of hormones. For parthenogenetic activation, cumulus-free oocytes were electroactivated in activation medium (280 mM mannitol, 0.01 mM CaCl2 and 0.05 mM MgCl2) with an electric pulse (1.0 kV/centimeter for 60 ms) using a BTX Electro-cell Manipulator (BTX, CA, USA). Immediately after electroactivation, the zygotes were cultured in porcine zygote medium 3 (PZM3) supplemented with 2 mmol/L 6-dimethylaminopurine (6-DMAP) for 4 h. Subsequently, the zygotes were cultured in PZM3 without 6-DMAP for 7 days. Hatched embryos were transferred to RNAlater and stored until RNA isolation.

### 2.3 Isolation and in vitro culture of pig fetal fibroblasts

Pig fetal fibroblasts (PFFs, mixed breed) were obtained from approximately 30-day-old fetuses after artificial insemination. The head, limbs, and internal organs were removed. The remaining tissue was minced and cultured in DMEM (Welgene, Korea) supplemented with 10% fetal bovine serum (FBS; collected and processed in the United States; Genedepot, TX, USA), 1 × Glutamax (Gibco), 0.1 mM β-mercaptoethanol (Gibco), and 1 × antibiotic/antimycotic (Gibco).

### 2.4 In vitro culture of pig embryonic stem-like cells

Pig embryonic stem-like (ES-like) cells were derived and cultured according to our previous studies [2]. The ES-like cell media medium of a 1:1 mixture of Dulbecco's modified Eagle's medium (DMEM) and Ham's F10 media containing 15% fetal bovine serum (FBS; collected and processed in the USA), 2 mM Glutamax, 0.1 mM β-mercaptoethanol, 1 ´ MEM nonessential amino acids, 1 ´ antibiotic–antimycotic (all from Gibco, USA), 40 ng/ml human recombinant stem cell factor (hrSCF; R&D Systems, USA) and 20 ng/ml human recombinant basic fibroblast growth factor (hrbFGF; R&D Systems). The medium was changed every 24 h, and all cells were cultured in humidified conditions with 5% CO2 at 37 °C. ES-like cells were subcultured every 5–7 days using pulled glass pipettes. Expanded colonies were detached from the feeder cells and dissociated into small clumps. These clumps were transferred to new feeder cells consisting of mitomycin-C-treated (Roche, Germany) mouse embryonic fibroblasts.

### 2.5 Formation of embryoid bodies from pig ES-like cells

Cultured ES-like cell colonies were detached from feeder cells, and the colonies were mechanically dissociated into small clumps. Suspension cultures of these clumps were obtained using the hanging-drop method for 5–6 days with ES-like cell media in the absence of cytokines. After hanging-drop culture, small clumps were aggregated and formed embryoid bodies.

### 2.6 Isolation and sequencing of miRNAs

miRNAs were isolated from samples using the mirVANA miRNA Isolation Kit following the manufacturer's instruction (Invitrogen, Carlsbad, CA, USA). After validation of extraction (RNA concentration, optical density ratio, 28S:18S ratio, etc.), adapters were applied to the RNA, and RNA libraries were prepared using NEXTFLEX® Small RNA-Seq Kit v3 for Illumina® Platforms. RNA sequencing was performed using HiSeq2500 (Illumina).

### 2.7 Analysis of the sequencing data

All of the program-based analysis was conducted by LifeGenomics. The quality of the reads was checked with the FastQC program. Reads without adapter sequences and with long lengths were trimmed out using Cutadapt [4]. All RNA reads were aligned and quantified with Bowtie [5], Samtools and HTSeq [6]. To evaluate the quality of the miRNA-seq data and utilize the information for other captured small RNAs, all RNAs were quantified as defined in ENSEMBL/GENCODE annotations [7]. For normalization of the miRNA read counts within samples, Tag Count Comparison (TCC) was used (an R package using for tag count comparison) [3]. All miRNA reads were categorized as reported or novel miRNAs using miRDeep2 based in miRBase [8,9].

### 2.8 Sample-specific miRNAs

To compare miRNA expression levels between samples, the expression levels of miRNAs were considered high when they were 2-fold higher in one sample than the other. Before the comparison, the average level in the BL samples was used as representative value. First, miRNAs that were expressed at a higher levels in a specific sample than all of the others were listed (sheet 3 of Dataset 2). Then, 72, 28, 19 and 39 miRNAs were sorted in BL, ES-like, PEB and PFF samples, respectively. Second, we compared pluripotent (BL and ES-like) and differentiated (PEB and PFF) samples. miRNAs with relatively high expression levels were listed. Conditions of selection are also described on sheet 3 of Dataset 2. The target genes of the sample-specific miRNAs were predicted with TargetScanHuman based on human data (sheet 1 of Dataset 3). GO enrichment and KEGG pathway analyses were conducted for the target gene data. Detection scores of each miRNA for each sample are provided in Dataset 3 (sheets 2 to 9). Levels 2, 4, 6, 8 and 10 were used for GO term counting.

## Ethics Statement

Animal care and experimental use of samples were approved by the Institutional Animal Care and Use Committee (IACUC) of Seoul National University (Approval no. SNU-140,328–2).

## Declaration of Competing Interest

The authors declare that they have no known competing financial interests or personal relationships which have, or could be perceived to have, influenced the work reported in this article.

## Appendix Supplementary materials

Image, application 1

Image, application 2

Image, application 3
